# Supplementary material for: Safety of Post-Transplant Cyclophosphamide-Based Prophylaxis in AML Patients with Pre-Existing Cardiac Morbidity Undergoing Allogeneic Hematopoietic Cell Transplantation
Source: Cancers (Basel). 2025 Sep 26;17(19):3128. doi: 10.3390/cancers17193128 (PMC12523613; doi:10.3390/cancers17193128)
Supplement: Supplementary file 1 [file cancers-17-03128-s001.zip › cancers-3857411-supplementary.pdf]

## SUPPLEMENTARY MATERIAL:

**Supplementary Table 1. Cause-specific hazard models distinguishing deaths directly attributable to cardiac events from deaths due to other causes**

| | Model | Censor | Event | Competing Event | HR (95%CI) | p.value |
|---|---|---|---|---|---|---|
| **Unbalanced** | **Competing risk** | Alive at last follow-up | CE-related death | Death not related to CE | 3.35 (0.84-13.30) | 0.086 |
| | **Global OS** | Alive at last follow-up | Any death | - | 1.17 (0.76-1.80) | 0.486 |
| | **CE OS** | Alive at last follow-up | CE-related death | - | 3.37 (0.84-13.5) | 0.086 |
| | **Other OS** | Alive at last follow-up | Death not related to CE | - | 1.07 (0.67-1.69) | 0.781 |
| **Matching** | **Competing risk** | Alive at last follow-up | CE-related death | Death not related to CE | 2.50 (0.60-10.40) | 0.197 |
| | **Global OS** | Alive at last follow-up | Any death | - | 1.19 (0.76-1.87) | 0.451 |
| | **CE OS** | Alive at last follow-up | CE-related death | - | 2.48 (0.59-10.38) | 0.195 |
| | **Other OS** | Alive at last follow-up | Death not related to CE | - | 1.11 (0.69-1.79) | 0.682 |

**Supplementary Table 2. Proportion and distribution of missing data across covariates used in the IPW model, as well as a comparison between imputed and non-imputed datasets.**

| Characteristic | Imputed<br>N = 461[1] | No imputed<br>N = 461[1] | p-value[2] |
|---|---|---|---|
| **HTA1** | | | >0.9 |
| No | 366 (79%) | 365 (79%) | |
| Si | 95 (21%) | 95 (21%) | |
| Missing | 0 | 1 | |
| **Age** | | | >0.9 |
| Mean (SD) | 52 (14) | 52 (14) | |
| Median (Q1, Q3) | 55 (43, 63) | 55 (43, 63) | |
| Min, Max | 17, 79 | 17, 79 | |
| **Intensity1** | | | >0.9 |
| MAC | 249 (54%) | 249 (54%) | |
| RIC | 212 (46%) | 212 (46%) | |
| **Status2** | | | >0.9 |
| RC | 414 (90%) | 414 (90%) | |
| R/R LMA | 47 (10%) | 47 (10%) | |
| **Donor** | | | >0.9 |
| MSD | 86 (19%) | 86 (19%) | |
| MUD | 77 (17%) | 77 (17%) | |
| MMUD | 30 (6.5%) | 30 (6.5%) | |
| HAPLO | 268 (58%) | 268 (58%) | |
| **HCT_mas3** | | | 0.8 |
| No | 380 (82%) | 365 (83%) | |
| Si | 81 (18%) | 73 (17%) | |
| Missing | 0 | 23 | |
| **DLP1** | | | >0.9 |
| No | 401 (87%) | 399 (87%) | |
| Si | 60 (13%) | 60 (13%) | |
| Missing | 0 | 2 | |
